# Organic electron transport materials

**DOI:** 10.3762/bjoc.20.60

**Published:** 2024-03-28

**Authors:** Joseph Cameron, Peter J Skabara

**Affiliations:** 1 School of Chemistry, University of Glasgow, University Avenue, Glasgow, G12 8QQ, UK

**Keywords:** acceptors, electron transport material, electron-deficient, n-type, organic semiconductors

There has been much interest in developing electron transport materials for organic semiconductor devices due to their potential to be applied in various technologies. For example, such materials can be used to improve charge balance in the emissive layer of an organic light-emitting diode, charge extraction in perovskite solar cells or used as the semiconductor layer of n-type organic field-effect transistors.

Typically, an electron transport material should have a high electron mobility and a low-lying lowest unoccupied molecular orbital (LUMO) relative to vacuum. This is achieved by using electron-deficient units containing highly electronegative functionalities such as cyano groups [1]. This is one of the key advantages of organic electron transport materials compared with inorganic materials – the molecular structures can be easily tuned to achieve desired characteristics. The diversity of materials used as electron transport layers is also an advantage, meaning organic materials can be used effectively in a range of applications. For example, while a non-fullerene acceptor material might not be suitable as an electron transport layer in OPVs due to dissolution of layers, it can used in perovskite solar cells where it can be successfully deposited through orthogonal processing.

One of the biggest challenges with n-type materials is air stability. This is explained by the redox potentials of water (−0.66 V vs standard calomel electrode (SCE)) and oxygen (+0.024 V vs SCE, +0.57 V vs SCE) [2–3]. Therefore, it has been observed that once the overpotential required for these redox reactions to progress is taken into consideration, a lowest unoccupied molecular orbital (LUMO) energy ≤ −4.0 eV (relative to vacuum) is required for air stable electron transport materials [2] and this is a common target for researchers developing these types of materials. In general, this restriction also applies to n-dopants, compounds that reduce electron transport materials, which are important for increasing the electrical conductivity of n-type materials. However, there have been elegant solutions developed to counteract this, where air-stable dimers can be used which react with acceptors to produce reducing radical species, capable of reducing organic electron transport materials with a low electron affinity [4–5].

It is not only in modifying the molecular structure to improve the electron accepting ability that there is innovation in new organic electron transport materials. One of the potential benefits of organic semiconductors is the ability to use solution-processing techniques, which are more sustainable than thermal evaporation which is wasteful and requires high vacuum and temperature. Moreover, organic electron transport layers do not typically need treatment at the high temperatures required for metal oxide formation for example. However, making materials solution-processable can result in a trade-off in device performance and materials should be applicable to orthogonal processing to allow for solution-based fabrication of multilayer devices. Additionally, researchers should consider minimising the environmental footprint of the solvents and materials used in these processes. These challenges require novel approaches but there have already been some inspired works. For example, Wolfe et al. showed cathode interlayer materials can be developed to be soluble in ethyl acetate, a green solvent, and solution-processed for PM6:Y6 bulk-heterojunction solar cells with power conversion efficiency > 13% [6]. In addition to orthogonal processing, it has been shown that treatment with base [7] or photo-crosslinking [8] can insolubilise solution-processed layers and allow for the construction of efficient, solution-processed devices.

It is exciting to see how much of the work establishing n-type organic semiconductor materials for OFETs, for example, is now being applied to emerging technologies such as organic electrochemical transistors. In this case, materials might be modified with polar side chains [9], instead of the use of alkyl groups [10], and this allows for improved ion transport. A similar strategy has been used to improve the doping of n-type thermoelectric materials, where polar side chains on fullerene materials allow for better miscibility of the dopant [11] and therefore improved electrical conductivity. The overlap of desired characteristics for materials used in OECTs and organic thermoelectrics shows that there is a lot to be gained from the design of new electron transport materials.

Whilst there are challenges still to be overcome in developing materials for emerging technologies, researchers working on these issues should take inspiration from the rapid progress that was achieved in the development of non-fullerene acceptor materials as a replacement for PC_61_BM and PC_71_BM in organic solar cells which revitalised the research field as a result. This serves as a reminder that careful molecular design and thorough examination of the performance of electron transport materials can lead to unprecedented performance and move towards enabling important technologies.

We are delighted to act as guest editors for a collection of papers tackling many different aspects relating to organic electron transport materials from leaders across the world from this research community. We hope that these excellent works can act as inspiration for researchers looking to develop new materials or implement these in devices. We give our thanks to all the authors for their great contributions to this Thematic Issue and we thank the staff from the Beilstein-Institut for their support.

Joseph Cameron and Peter J. Skabara

Glasgow, March 2024

## Data Availability

Data sharing is not applicable as no new data was generated or analyzed in this study.
